# Characterizing the effects of calcineurin/NFAT inhibitor treatment on gray and white matter microstructure in the beagle model of Alzheimer's disease

**DOI:** 10.1002/alz.092102

**Published:** 2025-01-09

**Authors:** Jessica A. Noche, Hamsanandini Radhakrishnan, Margo F. Ubele, Kathy Boaz, Jennifer L. Mefford, Erin D. Jones, Jessica A. Perpich, Beverly Meacham, Jeffrey Smiley, Stasia A. Bembenek Bailey, Laszlo G. Puskas, David K Powell, Lorena Sordo, Michael J. Phelan, Christopher M. Norris, Elizabeth Head, Craig EL Stark

**Affiliations:** ^1^ University of California, Irvine, Irvine, CA USA; ^2^ Penn FTD Center, University of Pennsylvania, Philadelphia, PA USA; ^3^ University of Kentucky, Lexington, KY USA; ^4^ University of Kentucky College of Medicine, Lexington, KY USA; ^5^ Aperus Pharma, Szeged Hungary; ^6^ The UC Irvine Institute for Memory Impairments and Neurological Disorders (UCI MIND), Irvine, CA USA; ^7^ University of Kentucky College of Medicine, Sanders‐Brown Center on Aging, Lexington, KY USA; ^8^ Department of Neurobiology and Behavior, University of California, Irvine, Irvine, CA USA

## Abstract

**Background:**

In the context of pathological aging and Alzheimer's disease (AD), the overexpression of calcineurin has been identified as a factor linked to astrocyte reactivity, neuronal death, and inflammation. This suggests that inhibiting calcineurin or downstream nuclear factor of activated T cells (NFAT) signaling could be a promising strategy for preventing or slowing down AD pathophysiology.

**Method:**

Baseline and annual MRI sessions including higher‐order diffusion‐weighted imaging was performed over four years on 43 dogs ranging from 5 to 8.7 years old (placebo n=14; tacrolimus n=15; Q134R n=14). Estimates from diffusion tensor and neurite orientation dispersion and density imaging (NODDI) models were assessed: orientation dispersion index (ODI), intracellular volume fraction (ICVF), isotropic volume fraction (ISOVF), fractional anisotropy (FA), mean diffusivity (MD), radial diffusivity (MD), axial diffusivity (AxD). We examined the percent changes from select gray matter regions of interest, including the posterior cingulate gyrus (PCG), orbital gyrus, caudate nucleus, and hippocampus, as well as voxel‐wise white matter microstructure across the brain.

**Result:**

Across all groups, the PCG showed a 1% decrease in ODI, whereas the orbital gyrus had 1% increases in ODI and in ICVF. The orbital gyrus also had a 2% increase in MD and RD and a 26% increase in ISOVF. The hippocampus had a 0.8% increase in ODI. The caudate nucleus showed numerous changes, including a substantial 36% increase in ISOVF and 1.5% decrease in FA. Only Q134R‐treated dogs showed a 0.6% increase in AxD, and tacrolimus‐treated dogs showed 0.7% and 0.9% increases to MD and RD, respectively. Whole brain white matter demonstrated an anterior‐posterior gradient to diffusion changes similar to that of human aging across all groups: decreasing FA, increasing MD, increasing RD, and decreasing AxD.

**Conclusion:**

Microstructural alterations within gray and white matter of canines parallel human aging. The study suggests that Q134R and tacrolimus differentially impact gray matter microstructural changes within the caudate nucleus. Future investigations involving cognitive outcomes and histological evaluations will be crucial to understanding the neurobiological underpinnings of these diffusion changes and the effects of chronic tacrolimus and Q134R treatment on AD pathology and cognition.

